# Prevalence and correlates of psychotropic drug use in Dutch nursing home patients with young‐onset dementia

**DOI:** 10.1002/gps.5116

**Published:** 2019-04-29

**Authors:** Ans J.M.J. Mulders, Sytse U. Zuidema, Renée Leeuwis, Hans Bor, Frans R.J. Verhey, Raymond T.C.M. Koopmans

**Affiliations:** ^1^ Department of Primary and Community Care, Centre for Family Medicine, Geriatric Care and Public Health Radboud University Nijmegen Medical Centre Nijmegen The Netherlands; ^2^ Archipel Care Group, Landrijt Centre for Specialized Care Eindhoven The Netherlands; ^3^ De Waalboog, “Joachim en Anna,” Centre for Specialized Geriatric Care Nijmegen The Netherlands; ^4^ Department of General Practice and Elderly Care Medicine, University of Groningen University Medical Centre Groningen Groningen The Netherlands; ^5^ Department of Psychiatry and Psychology/MUMC School for Mental Health and Neuroscience (MHeNS)/Alzheimer Centre Limburg Maastricht The Netherlands; ^6^ Radboudumc Alzheimer Centre Nijmegen The Netherlands

**Keywords:** long‐term care, nursing home, psychotropic drug use, young‐onset dementia

## Abstract

**Objectives:**

Neuropsychiatric symptoms (NPSs) in dementia patients in nursing homes are common and often treated with psychotropic drugs. Thus far, studies on psychotropic drug use (PDU) mainly focused on elderly patients with dementia. It is important to know the prevalence and correlates of PDU in patients with young‐onset dementia (YOD) to optimize NPS treatment and reduce excess PDU in this group.

**Design:**

The Behavior and Evolution in Young‐Onset Dementia (BEYOnD) study is a cross‐sectional study in 230 residents with YOD in eight nursing homes providing specialized YOD care. PDU was registered from medical charts. The influence of age, gender, severity, and type of dementia, NPS, and agitation on PDU was analyzed.

**Results:**

Eighty‐seven percent of the residents used psychotropic drugs, and 47% used two or more psychotropic drugs. No significant associations with age, gender, dementia subtype, and dementia severity were found. Depressive symptoms showed an association with the use of antidepressants, nighttime behavior with use of hypnotics, and verbal agitation with antipsychotics. Apathy was strongly associated with lower PDU.

**Conclusions:**

Nursing home patients with YOD have a high prevalence of PDU. The association with NPS raises questions of efficacy of these drugs in managing NPS. Patients with apathy had a lower chance of using psychotropics. More research on indications and effects of psychotropic drugs is needed, as well as on (nonpharmacological) alternatives for managing challenging behavior in YOD.

**Key points**

- Psychotropic drug use is very high in patients with young‐onset dementia in nursing homes.
- In almost half of the YOD patients, multiple psychotropic drugs are used.
- Apathy is strongly associated with less use of psychotropic drugs.

## 1 INTRODUCTION

Although dementia is typically regarded as a disease of old age, dementia also occurs at younger age. Young‐onset dementia (YOD) with first symptom onset before the age of 65^1^ is recognized as an important psychosocial and medical health problem with serious consequences for patients and their families.^2^, ^3^, ^4^

The prevalence^4^, ^5^, ^6^ of YOD has been estimated between 67 and 98 per 100 000 for the age of 45 to 65 years and 12 per 100 000 for the age of 30 to 44 years. In The Netherlands, it is estimated that there are approximately 12 000 people with YOD of whom approximately 2500 are residing in nursing homes.^7^

In patients with young onset as well as late‐onset dementia (LOD), Alzheimer's disease is the most common cause ranging from 30% to 34% in YOD. In patients with YOD, however, there are larger proportions of frontotemporal dementia (FTD), alcohol‐related dementia (AlcD), and other causes of cognitive deterioration^5^, ^8^ compared with those with LOD.

Dementia often comes with neuropsychiatric symptoms (NPSs), such as depression, agitation, or apathy. In YOD, research has already demonstrated that NPSs are frequent and burdensome^9^ and are important predictors for institutionalization.^10^ However, studies on frequency and type of NPS are scarce and show conflicting results. In community‐dwelling patients with young‐onset Alzheimer's disease (YO‐AD), these patients showed less NPS than a comparable group with LOad. 9, 11 However, in the total group of community‐dwelling patients, those with YOD had significantly greater levels of both hyperactive symptoms and apathy and significantly lower levels of mood symptoms.^10^ In institutionalized YOD patients, our study showed that significant NPS occurred in almost 90% of all patients, consisting most frequently of agitation (66%) and apathy (56%), where psychotic symptoms and depressive symptoms were less frequent (11% and 19%, respectively). The prevalence and severity of NPS were higher than in institutionalized LOD patients.^12^

Neuropsychiatric symptoms may be treated with psychotropic drugs, which have limited efficacy and frequent, severe side effects. Especially, the use of antipsychotic drugs in patients with dementia has been shown to increase the risk of death,^13^, ^14^ stroke,^15^, ^16^ and cardiac arrhythmias^17^ although this is not confirmed in a more recent meta‐analysis including randomized controlled trials.^18^ A recent Norwegian study in nursing home patients did not show an association of PDU with long‐term mortality risk.^19^ Less controversial is the occurrence of adverse effects such as parkinsonism,^20^ gait disturbances, and daytime sedation,^21^ which have been shown to negatively influence quality of life.^22^

All current medical guidelines on management of NPSs in dementia recommend psychosocial interventions as first‐line treatment of NPSs. Nonpharmacological approaches have been used successfully to reduce NPSs.^23^, ^24^ Despite these recent recommendations, psychotropic drugs are widely prescribed in dementia patients with NPSs. Studies regarding psychotropic drug use in nursing home patients with LOD found that 37% to 78% had at least one prescription of a psychotropic drug, 21% to 54% used antipsychotics, 15% to 31% sedatives, 9% to 38% anxiolytics, and 19% to 47% antidepressants.^25^

An association between specific NPSs and the use of different types of psychotropic drugs has been shown.^26^ Antipsychotic medication was more frequently prescribed in patients with at least one NPS^27^, ^28^ and more specifically in patients with psychosis, agitation, and nighttime behavior.^29^

Studies on PDU in YOD patients are scarce. Thus far, after a comprehensive search in PUBMED, only one study was found. In this study, in community‐dwelling YOD patients, PDU^30^ was as high as 52%. In a study with a comparable community‐dwelling LOD group, this was 28.8%, and increasing age and moderate to severe depressive symptoms were positively associated with the total use of psychotropic drugs.^31^ No studies were found on PDU in institutionalized YOD patients. In The Netherlands, the Dutch Knowledge Center for YOD, a cooperation of over 20 care organizations delivering specialized care for persons with YOD, provides a unique opportunity for research in residential care for YOD. The YOD special care units are usually part of (larger) nursing homes, delivering care for five to 25 YOD patients per unit, with a specially trained multidisciplinary nursing home staff and services for younger persons. In these nursing homes, integrated medical care is delivered by elderly care physicians,^32^ who are generally responsible for all drug prescriptions in the nursing home setting.

The aim of this study is (a) to gain insight into the point prevalence of psychotropic drug use in institutionalized patients with YOD and (b) to study the association between age, gender, dementia subtype, severity of dementia, NPSs, and the use of psychotropic drugs.

## 2 METHODS

### 2.1 Participants

This study is part of the Behavior and Evolution in Young‐Onset Dementia (BEYOnD) study.^33^ In this study, eight Dutch nursing homes with in total 21 specialized care units for YOD patients participated. Inclusion took place between 2005 and 2009. Patients were considered for inclusion provided that they (a) met the DSM‐IV‐TR criteria (American Psychiatric Association, 2000) for dementia (b) with documented dementia symptoms in their entire medical record before the age of 65 and (c) resided in a nursing home with specialized care for YOD and (d) the legal representative gave their informed consent on participation in the study. Only one representative declined the use of her husband's data for study purposes.

The study protocol is in accordance with the Declaration of Helsinki, with the Dutch legislation on medical research, and it is in agreement with the Conduct Health Research of the Dutch federation of Biomedical Scientific Societies. The study protocol was approved by the Regional Medical Ethics Review Committee Arnhem‐Nijmegen. The committee stated that, in accordance with Dutch legislation, the study can be performed without a review procedure by the committee because in the study, only observational data gathered by nursing staff as part of their daily work were collected.

### 2.2 Data collection

Baseline characteristics such as age, gender, marital status, and time of institutionalization were retrieved from patients' charts.

The diagnosis and dementia subtype were retrieved from the medical record. The type of dementia was recorded as stated in the medical record, in the original diagnostic letter from the physician that established the diagnosis. Most patients had a comprehensive medical work‐up determining the type of dementia, including neurological and neuropsychological tests and imaging at a memory clinic. If this was not the case, then diagnosis was recorded as *not otherwise specified*.

The types of dementia were categorized into Alzheimer's dementia (AD), vascular dementia (VaD), FTD, AlcD, and “other causes” of dementia, including Lewy body dementia (LBD), Huntington's disease, dementia caused by acquired brain injury, and/or encephalopathy. On the day of inclusion, vocational nurses specifically assigned for individual patients were interviewed by the researcher (AM, elderly care physician) and a research assistant (psychologist) to collect the following assessments.

### 2.3 Assessments

The actual prescription of psychotropic drugs on the day of assessment was retrieved from the medical chart, using the Anatomical Therapeutical Chemical Classification^34^ and grouped into antipsychotics (N05A), anxiolytics (N05B), hypnotics/sedatives (N05C), antidepressants (N06A), and anti‐epileptics. All psychotropic drug use was registered, regular as well as Pro Re Nata (PRN) prescriptions. A PRN prescription indicates the possible administration as needed but is not necessarily administered at the assessment day.

The following data were collected for correlates. NPSs were assessed using the Neuropsychiatric Inventory—Nursing Home (NPI‐NH) version, Dutch version.^35^ The NPI‐NH includes 12 NPSs: delusions, hallucinations, agitation, depression, anxiety, euphoria/elation, apathy/indifference, disinhibition, irritability, aberrant motor behavior, nighttime disturbances, and appetite/eating change. The frequency and severity of each symptom are rated on a four‐point (1‐4) and three‐point (1‐3) Likert scale, respectively.

Agitation was assessed in more detail using the Cohen‐Mansfield Agitation Inventory (CMAI), Dutch version.^36^, ^37^ The CMAI assesses 29 types of agitated behavior. The frequency of each symptom is rated on a seven‐point scale ranging from 1 (*never*) to 7 (*several times an hour*). The CMAI has a well‐established (test‐retest and interrater) reliability and validity^38^ in nursing home patients.

Severity of the dementia was assessed using the Global Deterioration Scale,^39^ which consists of a seven‐point scale ranging from 1 (*no cognitive decline*) to 7 (*very severe cognitive decline*). GDS scores of 4 to 6 denote moderate, moderately severe, and severe cognitive decline, respectively.

### 2.4 Analysis

In the analysis of NPI‐NH scores, frequency and severity scores were multiplied, and each individual NPS was considered clinically relevant if the frequency × severity score was more than or equal to 4, for comparability with previous studies on NPS in dementia.^40^, ^41^ Six NPI‐NH items were grouped into the following categories: psychosis (hallucinations and delusions) and agitation (agitation, disinhibition, irritability, and aberrant motor behavior).^42^ For inclusion in either category, at least one separate symptom had to be clinically relevant. Depression, anxiety, apathy, nighttime behavior, eating changes, and euphoria were analyzed separately.

Clinically relevant agitation as measured with the CMAI was defined as behavior occurring at least once a week or more (frequency score ≥ 3). CMAI items were categorized into physically nonaggressive behavior, physically aggressive behavior, and verbal agitated behavior.^43^

Binomial logistic regression was applied with regular psychotropic drug use (total drug use and use of individual drug types, excluding anti‐epileptics because the indication for the prescription could not be verified) as the dependent variable and age (in 5‐year groups), gender, severity of dementia, type of dementia, and NPSs as independent variables. Two separate models were analyzed: one model with NPI‐NH symptoms and the other model with CMAI symptoms as independent variables for NPS. We accepted *p* < .05 as significant.

## 3 RESULTS

### 3.1 Patients' characteristics

Two hundred thirty YOD patients met the inclusion criteria. Of five patients, data on NPS were incomplete, resulting in inclusion of 225 patients. Patients had a mean age of 60.1 years (SD 7.3); most of the patients had a GDS stage 6, and 53% were male (Table 1).

**Table 1:** Patients' characteristics

Age mean, y	60.1 (range 39‐78)
<44 years	11 (5%)
45–54	43 (19%)
55–64	115 (51%)
>65	56 (25%)
Gender (% male)	n (%), 120 (53%)
Dementia stage (GDS)	
GDS ≤4 (mild to moderate dementia)	39 (17%)
GDS 5 (moderately severe dementia)	55 (24%)
GDS 6 (severe dementia)	68 (30%)
GDS 7 (very severe dementia)	63 (28%)
Type of dementia	
Alzheimer's dementia	72 (32%)
Vascular dementia	29 (13%)
Frontotemporal dementia	36 (16%)
Alcohol‐related dementia	40 (18%)
Dementia NOS and other types of dementia	48 (21%)
Neuropsychiatric symptoms	
NPI‐NH any significant symptom^a^	202 (89.8%; CI, 85.1‐93.1)
Two or more significant symptoms	167 (74.2%; CI, 68.1‐79.5)
NPI‐NH agitation cluster^b^	149(66.2%; CI, 59.8‐72.1)
NPI‐NH psychosis cluster^c^	25 (11.1%; CI, 7.6‐15.9)
CMAI any significant symptom^d^	197 (87.6%; CI, 82.6‐91.2)

Abbreviations: CI, confidence interval; CMAI, Cohen‐Mansfield Agitation Inventory; GDS, global deterioration scale; GRAD, Guidelines for the Rating of Awareness Deficits; NOS, not otherwise specified; NPI‐NH, Neuropsychiatric Inventory—Nursing Home.

a Any item with frequency (F) × severity (S) score more than or equal to 4.

b One or more significant symptom(s) of agitation/aggression, disinhibition, irritability, and/or aberrant motor behavior.

c One or both significant symptoms of delusions and hallucinations.

d Any item occurring once a week or more.

### 3.2 Prevalence of psychotropic drug use

Over 80% of the patients used any regular prescribed psychotropic drug (Table 2). In addition, antidementia drugs were used by 18 patients (8.0%), and anti‐epileptic drugs were used by 26% of the patients; half of these patients had a diagnosis of epilepsy in their medical record.

**Table 2:** Prevalence of psychotropic drug use among patients (n = 225)

Type of Medication (ATC Code)	Regular Prescription		Prescription of Medication PRN	
	n	(% of total)	n	(%)
Any psychotropic drug	183	(81.3)	60	(26.)
Antipsychotic drugs (N05A)	114	(50.7)	11	(4.9)
Anxiolytic drugs (N05B)	69	(30.7)	31	(13.8)
Hypnotics (N05C)	40	(17.8)	14	(6.2)
Antidepressant drugs (N06A)	111	(49.3)	0	(0)
Single drug use				
Only antipsychotic	25	(11.1)		
Only anxiolytic	9	(4.0)		
Only hypnotics	6	(2.7)		
Only antidepressant	37	(16.4)		
Total single drug use	77	(34.2)		
Combination of two psychotropic drugs^a^				
Antipsychotic + anxiolytic	17	(6.7)		
Antipsychotic + hypnotics	5	(2.2)		
Antipsychotic + antidepressant	28	(12.4)		
Anxiolytic + hypnotics	7	(3.1)		
Anxiolytics + antidepressant	8	(3.6)		
Antidepressant drugs + hypnotics	2	(0.9)		
Total use of two drugs	67	(29.8)		
Combination of three or more drugs^a^				
Antipsychotic + anxiolytic + antidepressant	19	(8.4)		
Antipsychotic + antidepressant drugs + hypnotics	11	(4.8)		
Antipsychotic + anxiolytic + hypnotics	3	(1.3)		
Antipsychotic + anxiolytic + antidepressant + hypnotics	6	(2.7)		
Total use of three or more drugs	39	(17.3)		

a Combinations of psychotropic drugs, except antidementia drugs, and anti‐epileptic drugs.

One third of the patients used one psychotropic drug; almost half of the patients used two or more, and 17% used three or more psychotropic drugs (antidementia drugs and anti‐epileptics were excluded in the combinations).

Antipsychotics, anxiolytics, and antidepressant drugs were the most frequently prescribed drugs. The most prevalent combinations of regularly prescribed drugs were an antipsychotic with an antidepressant or with an anxiolytic drug (Table 2).

### 3.3 Correlates of psychotropic drug use

Results of the multivariate logistic regression analysis for correlates of PDU are represented in Tables 3 and 4. Male gender was significantly associated with higher PDU (OR 2.27, *p* = .026, and or 2.14, *p* = .035, respectively, for the two models). No significant associations between age and PDU were found. Dementia subtype and dementia severity were also not significantly associated with total PDU. However, anxiolytic drugs were less frequently prescribed in patients with GDS stage less than or equal to 4 (or 0.12, *p* = .002, and/or 0.16, *p* = .007, respectively, for the two models), and antipsychotics were more frequently prescribed in non‐Alzheimer's type dementia (or 2.07, *p* = .045) in the NPI model.

**Table 3:** Association between patient characteristics, NPS (NPI‐NH), and psychotropic drug use in Dutch nursing home patients with young‐onset dementia

	Total Psychotropic Drugs			Antipsychotic Drugs			Anxiolytic Drugs			Antidepressant Drugs			Hypnotics		
	OR	95% CI^b^		OR	95% CI^b^		OR	95% CI^b^		OR	95% CI^b^		OR	95% CI^b^	
Age	1.00	0.95	1.01	1.00	0.96	1.05	1.00	0.96	1.05	1.01	0.97	1.05	0.98	0.93	1.04
Gender (male)	**2.27** a	**1.10**	**4.69**	1.460	0.83	2.56	0.88	0.48	1.27	1.50	0.86	2.61	0.53	0.24	1.14
GDS 2‐5 (GDS 6‐7 reference)	0.66	0.28	1.55	1.16	0.60	2.25	**0.42** a	**0.20**	**0.87**	1.02	0.53	1.96	0.55	0.22	1.36
Non‐Alzheimer's type (Alzheimer's type reference)	0.95	0.39	2.32	**2.07** a	**1.02**	**4.20**	1.04	0.50	2.19	0.76	0.39	1.52	0.70	0.28	1.76
NPI psychosis cluster	0.71	0.24	2.18	1.89	0.75	4.77	1.53	0.60	3.86	0.64	0.27	1.54	1.12	0.35	3.56
Agitation cluster	2.67	0.74	9.65	2.03	0.60	6.95	1.30	0.52	3.29	0.94	0.41	2.18	1.08	0.39	3.02
Depression	1.84	0.63	5.41	1.90	0.68	5.33	1.11	0.50	2.46	**2.30** a	**1.10**	**4.83**	0.32	0.10	1.10
Anxiety	0.75	0.25	2.28	0.86	0.27	2.75	1.24	0.53	2.92	0.71	0.31	1.64	1.18	0.39	3.62
Apathy	**0.27** a	**0.08**	**0.88**	0.72	0.35	1.49	0.62	0.29	1.33	0.78	0.38	1.59	**0.35** a	**0.14**	**0.90**
Nighttime behavior	2.13	0.46	9.87	2.47	0.97	6.31	**3.08** a	**1.25**	**7.61**	0.57	0.24	1.40	**6.71** a	**2.43**	**18.51**

Abbreviation: OR, odds ratio.

a If value 1 is not included in the 95% confidence interval, the *p* value is significant (*p* < .05); these results are printed in bold.

b 95% confidence interval (lower boundary, upper boundary).

**Table 4:** Association between patient characteristics, agitation (CMAI), and psychotropic drug use in Dutch nursing home patients with young‐onset dementia

	Total Psychotropic Drugs			Antipsychotic Drugs			Anxiolytic Drugs			Antidepressant Drugs			Hypnotics		
	OR	99% CI^d^		OR	99% CI^d^		OR	99% CI^d^		OR	99% CI^d^		OR	99% CI^d^	
Age	1.00	0.95	1.05	1.00	0.96	1.05	1.00	0.95	1.04	1.01	0.97	1.05	0.97	0.92	1.03
Gender (male)	**2.14** e	**1.05**	**4.34**	1.61	0.91	2.83	0.96	0.53	1.73	1.42	0.82	2.44	0.69	0.34	1.40
GDS 2‐5 (GDS 6‐7 reference)	0.90	0.39	2.12	1.15	0.57	2.31	0.51	0.24	1.07	1.20	0.62	2.33	0.71	0.29	1.72
Non‐Alzheimer's type (Alzheimer's type reference)	0.88	0.38	2.03	1.63	0.83	3.20	0.82	0.41	1.63	0.74	0.39	1.42	0.59	0.26	1.34
CMAI physically nonaggressive behavior^a^	1.22	.057	2.60	1.25	0.67	2.33	1.39	0.71	2.72	1.53	0.84	2.79	1.42	0.63	3.20
Physically aggressive behavior^b^	1.48	0.69	3.17	1.86	0.98	3.53	1.55	0.77	3.12	0.74	0.40	1.36	1.06	0.47	2.40
Verbally agitated behavior^c^	1.51	0.70	3.24	**2.54** e	**1.39**	**4.65**	1.28	0.67	2.44	1.44	0.80	2.59	1.11	0.52	2.36

Abbreviation: OR, odds ratio.

a Physically nonaggressive behavior (pacing, aimless wandering, hiding things, hoarding things, trying to get to a different place, handling things inappropriately, general restlessness, inappropriate dressing, or undressing).

b Physically aggressive behavior (hitting, pushing, scratching, cursing or verbal aggression, grabbing, screaming, spitting, and strange noises).

c Verbally agitated behavior (constant unwarranted request for attention/help, complaining, repetitive sentences or questions, and negativity).^43^

d 95% confidence interval (lower boundary, upper boundary).

e If value 1 is not included in the 95% confidence interval, the *p* value is significant (*p* < .05).

Of the NPSs, as measured by the NPI‐NH, depression was associated with the use of antidepressants (or 2.30, *p* = .27); apathy was negatively associated with psychotropic drugs in general (or 0.16, *p* = .008) and with the use of hypnotics (or 0.24, *p* = .010). Nighttime behavior was positively associated with the use of hypnotics (or 8.72, *p* = .000).

In the analysis of the associations with agitation, as measured by the CMAI, verbal agitation was associated with a higher risk of antipsychotic drug use (or 2.54, *p* = .002).

## 4 DISCUSSION

This study is to our knowledge the first to describe the prevalence and patient‐related correlations of psychotropic drug use of institutionalized YOD patients in nursing homes. Over 80% of these YOD patients used at least one psychotropic drug, of which antipsychotics and antidepressants were each used by 50% of the patients. Almost half of the users had a combination of two or more psychotropic drugs.

Male patients had a two times higher risk of using psychotropic drugs. Dementia severity measured by GDS showed that mild to moderate dementia was associated with more use of anxiolytics than patients in a more severe stage. Non‐AD type was associated with a higher risk of using antipsychotics. The latter two findings should be interpreted with caution since these effects only reached statistical significance in the NPI model. Patients with depression had a higher risk of using antidepressants, verbal agitation resulted in a greater risk of using antipsychotics, and nighttime behavior was positively associated with the use of anxiolytics and sedatives.

Apathy showed a strongly negative correlation with use of psychotropic drugs overall and sedatives in particular.

The prevalence rates of psychotropic drug use we found in this study are slightly higher or in the same range compared with LOD patients in nursing homes.^25^, ^26^, ^44^ Several studies found that lower age was associated with higher rates of (multiple) psychotropic drug use compared with older patients.^26^, ^27^, ^29^, ^45^, ^46^ We found a lower prevalence of PRN‐prescribed antipsychotics, although we found a higher PRN prescription of anxiolytic drugs compared with studies with LOD patients.^29^, ^45^, ^47^

Data on PDU in YOD patients are scarce. Some results are available from studies in community‐dwelling YOD patients. In one study, PDU was also high^30^ but not as high as in the institutionalized YOD population. In another part of the same study, it was demonstrated that the presence of NPS was a predictor for institutionalization^10^ and the prevalence of NPS was substantially higher in the institutionalized group compared with the community‐dwelling group. Since the presence and severity of NPS may be a predictor for PDU, this may be an explanation for the higher use of PD in institutionalized YOD patients. On the other hand, this raises the question of efficacy of psychotropic drugs for the management of NPS. Apparently, the NPSs are still present but may have been mitigated by the drugs to a more acceptable level. The difference in PDU may be due not only to differences in people with YOD or LOD but also to differences in prescribing patterns in different countries or to the difference in underlying diagnoses in the two groups; eg, AlcD and Huntington's disease are known to result in relatively high PDU.^48^, ^49^ Another explanation may be the higher impact of agitated or aggressive behavior on staff, thus leading to a demand for the prescription of psychotropic drugs.^50^

We found an association between male gender and psychotropic drug use in general. In literature, we find contradicting results on the association of PDU with gender. In some LOD studies, psychotropic drugs were positively associated with male gender^44^, ^46^; other studies found no correlation with gender^28^, ^29^ or found that females are more likely to be prescribed psychotropic drugs.^26^, ^45^

Dementia severity in our study was not significantly associated with PDU; only the use of anxiolytics showed a significant association with dementia severity. This is in line with previous LOD studies^26^, ^29^, ^51^ finding no association with PDU in general. Assuming that NPSs are the most important indication for PDU, the variation in NPS, with some symptoms increasing and other symptoms subsiding with advancing dementia,^52^, ^53^ may explain the relatively stable use of PD during the course of the disease.

In concordance with a Norwegian study,^28^ there were significant associations between the different groups of drugs and specific NPSs in the case of depressive symptoms and the use of antidepressants, nighttime behaviors and the use of anxiolytics or hypnotics, and verbal agitation with the use of antipsychotics.

In contrast to the aforementioned study, we found that patients with apathy were much less likely to use psychotropic drugs in general or sedatives in particular. This finding is in line with the current understanding that apathy occurs frequently in dementia as a separate NPS requiring appropriate attention^54^, ^55^ and does not have to be the result of (excess) psychotropic drug use in nursing homes. Apathy in YOD has an important impact on patients and their caregivers,^10^ and to influence, this may be regarded as one of the most important challenges in informal and professional care for persons with YOD.

One of the major strengths of our study is that we had access to a relatively large group of institutionalized YOD patients, residing in comparable care settings with availability of complete medical records including all prescribed drugs.

There are also some limitations to this study. First, the drug dose was not taken into account. Therefore, it was not possible to evaluate the dosage. Due to absence of data on the actual administration of PRN prescriptions, PDU may be underestimated. Furthermore, reasons for drug prescription, the severity of the symptoms when the drugs were first administered, and the duration of usage were not available for analysis, so only assumptions can be made based on concomitant existence of NPS and the use of drugs. We used the type of dementia as a determinant of psychotropic drug use, but the type of dementia is based on the clinical diagnosis, which may not be accurate in some patients.

The selection of patients in specialized YOD care units may result in bias. In the YOD‐specialized care units, a skilled multidisciplinary staff and a comprehensive program for NPS management are key components of care. This may have resulted in a selection of patients with more challenging behavior in these care units, where patients with less NPS may be more likely to be taken care of at home or in a regular care facility nearby. On the other hand, more skilled teams and better equipped facilities may result in less psychotropic drug use, compared with care for YOD patients in less adapted facilities.

Because of the amount of variables in the analysis, there is a chance of type I error. However, for this first explorative study on NPS in institutionalized YOD patients, we chose to keep relevant variables in the model, with respect to the rule of thumb regarding the amount of events per variable. In case of total psychotropic drug use and the use of antipsychotics, the associations shown are strong and reliable. However, in the case of anxiolytics and hypnotics, groups were smaller, and therefore, the results should be interpreted with more caution.

## 5 CONCLUSION

In conclusion, we demonstrated that PDU in institutionalized YOD patients is very high and also, the use of multiple psychotropic drugs is widespread. A recent study showed that in only a minority of cases, the prescriptions psychotropic drugs for NPS in dementia were completely appropriate.^56^ It is likely that this is also the case in prescriptions for YOD patients. These results show the need for implementation of guidelines on psychotropic drug use and for nonpharmacological options to reduce the prevalence of NPS in specialized care units.

On the basis of the results of this first study to describe the prevalence and determinants of psychotropic drug use of patients with YOD in nursing homes, we can conclude that more research is needed on psychotropic drug use in patients with YOD, especially the changes in use over time, and evaluation of effectiveness of nonpharmacological interventions for NPS such as training of nurses, person‐centered care, and individualized structured day programs. Monitoring the effect of implementation of recent guidelines^57^ for adequate prescription of psychotropic drug use specifically in the YOD population may provide additional information on specific requirements and challenges this group brings about in nursing homes.

### DATA AVAILABILITY STATEMENT

Data available on request from the authors. The data that support the findings of this study are available from the corresponding author upon reasonable request.

## FUNDING INFORMATION

This study was funded by the Archipel Care Group in Eindhoven, The Netherlands, Radboud University Nijmegen, and Stichting Wetenschaps Bevordering Verpleeghuizen (SWBV).

## CONFLICT OF INTEREST

None declared.

## References

[1] Alzheimer's Association . Early Onset Dementia: A National Challenge, A Future Crisis. 2006 Available at: http://www.alz.org/national/documents/report_earlyonset_full.pdf; last accessed 18 November 2018.

[2] Bakker C , de Vugt ME , Vernooij‐Dassen M , van Vliet D , Verhey FRJ , Koopmans RTCM . Needs in early onset dementia: a qualitative case from the NeedYD study. Am J Alzheimers Dis. 2010;25(8):634‐640. 10.1177/1533317510385811 PMC10845426 21131669

[3] Mendez MF . The accurate diagnosis of early‐onset dementia. Int J Psychiatry Med. 2006;36(4):401‐412. 17407994 10.2190/Q6J4-R143-P630-KW41

[4] Sampson EL , Warren JD , Rossor MN . Young onset dementia. Postgrad Med J. 2004;80(941):125‐139. 15016933 10.1136/pgmj.2003.011171 PMC1742955

[5] Harvey RJ , Skelton‐Robinson M , Rossor MN . The prevalence and causes of dementia in people under the age of 65 years. J Neurol Neurosurg Psychiatry. 2003;74(9):1206‐1209. 12933919 10.1136/jnnp.74.9.1206 PMC1738690

[6] Kelley BJ , Boeve BF , Josephs KA . Young‐onset dementia: demographic and etiologic characteristics of 235 patients. Arch Neurol. 2008;65(11):1502‐1508. 19001170 10.1001/archneur.65.11.1502

[7] Statistiek CBvd . Monitor langdurige zorg. 2014; http://mlzstatline.cbs.nl/Statweb/publication/?DM=SLNL&PA=40010NED&D1=2&D2=0&D3=0‐1,5,9&D4=0‐2,7,9,15,46,l&D5=0&D6=6–10&HDR=G1,T,G5,G2&STB=G3,G4&VW=T. Accessed 13 April 2017.

[8] Picard C , Pasquier F , Martinaud O , Hannequin D , Godefroy O . Early onset dementia: characteristics in a large cohort from academic memory clinics. Alzheimer Dis Assoc Disord. 2011;25(3):203‐205. 21192236 10.1097/WAD.0b013e3182056be7

[9] van Vliet D , de Vugt ME , Aalten P , et al. Prevalence of neuropsychiatric symptoms in young‐onset compared to late‐onset Alzheimer's disease—part 1: findings of the two‐year longitudinal NeedYD‐study. Dement Geriatr Cogn Disord. 2012;34(5–6):319‐327. 23208452 10.1159/000342824

[10] Bakker C , de Vugt ME , van Vliet D , et al. Predictors of the time to institutionalization in young‐ versus late‐onset dementia: results from the needs in young onset dementia (NeedYD) study. J Am Med Dir Assoc. 2012;14(4):248‐253. 23123009 10.1016/j.jamda.2012.09.011

[11] Toyota Y , Ikeda M , Shinagawa S , et al. Comparison of behavioral and psychological symptoms in early‐onset and late‐onset Alzheimer's disease. Int J Geriatr Psychiatry. 2007;22(9):896‐901. 17343292 10.1002/gps.1760

[12] Mulders AJ , Fick IW , Bor H , Verhey FR , Zuidema SU , Koopmans RT . Prevalence and correlates of neuropsychiatric symptoms in nursing home patients with young‐onset dementia: the BEYOnD study. J Am Med Dir Assoc. 2016;17(6):495‐500. 26944910 10.1016/j.jamda.2016.01.002

[13] Schneider LS , Dagerman KS , Insel P . Risk of death with atypical antipsychotic drug treatment for dementia: meta‐analysis of randomized placebo‐controlled trials. JAMA. 2005;294(15):1934‐1943. 16234500 10.1001/jama.294.15.1934

[14] Kales HC , Kim HM , Zivin K , et al. Risk of mortality among individual antipsychotics in patients with dementia. Am J Psychiatry. 2012;169(1):71‐79. 22193526 10.1176/appi.ajp.2011.11030347 PMC4269551

[15] Kleijer BC , Heerdink ER , Egberts TC , Jansen PA , van Marum RJ . Antipsychotic drug use and the risk of venous thromboembolism in elderly patients. J Clin Psychopharmacol. 2010;30(5):526‐530. 20814323 10.1097/JCP.0b013e3181f0e87d

[16] Rochon PA , Normand SL , Gomes T , et al. Antipsychotic therapy and short‐term serious events in older adults with dementia. Arch Intern Med. 2008;168(10):1090‐1096. 18504337 10.1001/archinte.168.10.1090

[17] Ray WA , Chung CP , Murray KT , Hall K , Stein CM . Atypical antipsychotic drugs and the risk of sudden cardiac death. N Engl J Med. 2009;360(3):225‐235. 19144938 10.1056/NEJMoa0806994 PMC2713724

[18] Hulshof TA , Zuidema SU , Ostelo RW , Luijendijk HJ . The mortality risk of conventional antipsychotics in elderly patients: a systematic review and meta‐analysis of randomized placebo‐controlled trials. J Am Med Dir Assoc. 2015;16(10):817‐824. 25933724 10.1016/j.jamda.2015.03.015

[19] Selbaek G , Aarsland D , Ballard C , et al. Antipsychotic drug use is not associated with long‐term mortality risk in Norwegian nursing home patients. J Am Med Dir Assoc. 2016;17(5):464 e461‐464 e467. 10.1016/j.jamda.2016.01.016 26935533

[20] Schouten HJ , Knol W , Egberts TC , Schobben AF , Jansen PA , van Marum RJ . Quality of life of elderly patients with antipsychotic‐induced parkinsonism: a cross‐sectional study. J Am Med Dir Assoc. 2012;13(1):82 e81‐82 e85. 10.1016/j.jamda.2010.12.003 21450229

[21] Schneider LS , Dagerman K , Insel PS . Efficacy and adverse effects of atypical antipsychotics for dementia: meta‐analysis of randomized, placebo‐controlled trials. Am J Geriatr Psychiatry. 2006;14(3):191‐210. 16505124 10.1097/01.JGP.0000200589.01396.6d

[22] Wetzels RB , Zuidema SU , de Jonghe JF , Verhey FR , Koopmans RT . Determinants of quality of life in nursing home residents with dementia. Dement Geriatr Cogn Disord. 2010;29(3):189‐197. 20215750 10.1159/000280437

[23] O'Connor DW , Ames D , Gardner B , King M . Psychosocial treatments of psychological symptoms in dementia: a systematic review of reports meeting quality standards. Int Psychogeriatr. 2009;21(2):241‐251. 19138459 10.1017/S1041610208008223

[24] Sloane PD , Hoeffer B , Mitchell CM , et al. Effect of person‐centered showering and the towel bath on bathing‐associated aggression, agitation, and discomfort in nursing home residents with dementia: a randomized, controlled trial. J Am Geriatr Soc. 2004;52(11):1795‐1804. 15507054 10.1111/j.1532-5415.2004.52501.x

[25] Janus SI , van Manen JG , Mj IJ , Zuidema SU . Psychotropic drug prescriptions in western European nursing homes. Int Psychogeriatr. 2016;28(11):1775‐1790. 27469071 10.1017/S1041610216001150

[26] Gulla C , Selbaek G , Flo E , Kjome R , Kirkevold O , Husebo BS . Multi‐psychotropic drug prescription and the association to neuropsychiatric symptoms in three Norwegian nursing home cohorts between 2004 and 2011. BMC Geriatr. 2016;16(1):115. 27245665 10.1186/s12877-016-0287-1 PMC4888256

[27] Lindesay J , Matthews R , Jagger C . Factors associated with antipsychotic drug use in residential care: changes between 1990 and 1997. Int J Geriatr Psychiatry. 2003;18(6):511‐519. 12789672 10.1002/gps.871

[28] Selbaek G , Kirkevold O , Engedal K . The course of psychiatric and behavioral symptoms and the use of psychotropic medication in patients with dementia in Norwegian nursing homes—a 12‐month follow‐up study. Am J Geriatr Psychiatry. 2008;16(7):528‐536. 18591573 10.1097/JGP.0b013e318167ae76

[29] Nijk RM , Zuidema SU , Koopmans RT . Prevalence and correlates of psychotropic drug use in Dutch nursing‐home patients with dementia. Int Psychogeriatr. 2009;21(3):485‐493. 19368752 10.1017/S1041610209008916

[30] Koopmans RT , Reinders R , van Vliet D , et al. Prevalence and correlates of psychotropic drug use in community‐dwelling people with young‐onset dementia: the NeedYD‐study. Int Psychogeriatr. 2014;26(12):1983‐1989. 24411220 10.1017/S1041610213002330

[31] Chan DC , Kasper JD , Black BS , Rabins PV . Clinical diagnosis of dementia, not behavioral and psychologic symptoms, is associated with psychotropic drug use in community‐dwelling elders classified as having dementia. J Geriatr Psychiatry Neurol. 2007;20(2):100‐106. 17548780 10.1177/0891988706298628

[32] Koopmans RT , Lavrijsen JC , Hoek JF , Went PB , Schols JM . Dutch elderly care physician: a new generation of nursing home physician specialists. J Am Geriatr Soc. 2010;58(9):1807‐1809. 20863347 10.1111/j.1532-5415.2010.03043.x

[33] Mulders AJ , Zuidema SU , Verhey FR , Koopmans RT . Characteristics of institutionalized young onset dementia patients—the BEYOnD study. Int Psychogeriatr. 2014;26(12):1973‐1981. 25295790 10.1017/S1041610214001859

[34] Nordic Council on Medicines . Guidelines for ATC Classification WHO Collaborating Center for Drugs Statistics Methodology. Oslo (Norway): Nordic Councilon Medicines; 1990.

[35] Kat MG , de Jonghe JF , Aalten P , et al. Neuropsychiatric symptoms of dementia: Psychometric aspects of the Dutch Neuropsychiatric Inventory (NPI). Tijdschr Gerontol Geriatr. 2002;33:150‐155. Dutch. 12378786

[36] Cohen‐Mansfield J , Billig N . Agitated behaviors in the elderly. I. A conceptual review. J Am Geriatr Soc. 1986;34:711‐721. 3531296 10.1111/j.1532-5415.1986.tb04302.x

[37] de Jonghe JF , Kat MG . Factor structure and validity of the Dutch version of the Cohen‐Mansfield Agitation Inventory (CMAI‐D). J Am Geriatr Soc. 1996;44:888‐889. 8675952 10.1111/j.1532-5415.1996.tb03762.x

[38] Miller RJ , Snowdon J , Vaughan R . The use of the Cohen‐Mansfield Agitation Inventory in the assessment of behavioral disorders in nursing homes. J Am Geriatr Soc. 1995;43:546‐549. 7730539 10.1111/j.1532-5415.1995.tb06104.x

[39] Reisberg B , Ferris SH , de Leon MJ , Crook T . The Global Deterioration Scale for assessment of primary degenerative dementia. Am J Psychiatry. 1982;139:1136‐1139. 7114305 10.1176/ajp.139.9.1136

[40] Wood S , Cummings JL , Hsu MA , et al. The use of the neuropsychiatric inventory in nursing home residents. Characterization and measurement. Am J Geriatr Psychiatry. 2000;8(1):75‐83. 10648298 10.1097/00019442-200002000-00010

[41] Margallo‐Lana M , Swann A , O'Brien J , et al. Prevalence and pharmacological management of behavioural and psychological symptoms amongst dementia sufferers living in care environments. Int J Geriatr Psychiatry. 2001;16(1):39‐44. 11180484 10.1002/1099-1166(200101)16:1<39::aid-gps269>3.0.co;2-f

[42] Zuidema SU , de Jonghe JF , Verhey FR , Koopmans RT . Neuropsychiatric symptoms in nursing home patients: factor structure invariance of the Dutch nursing home version of the neuropsychiatric inventory in different stages of dementia. Dement Geriatr Cogn Disord. 2007;24(3):169‐176. 17641527 10.1159/000105603

[43] Zuidema SU , de Jonghe JF , Verhey FR , Koopmans RT . Agitation in Dutch institutionalized patients with dementia: factor analysis of the Dutch version of the Cohen‐Mansfield Agitation Inventory. Dement Geriatr Cogn Disord. 2007;23(1):35‐41. 17077631 10.1159/000096681

[44] Nobili A , Pasina L , Trevisan S , et al. Use and misuse of antipsychotic drugs in patients with dementia in Alzheimer special care units. Int Clin Psychopharmacol. 2009;24(2):97‐104. 21456106 10.1097/yic.0b013e328323aaf0

[45] Draper B , Brodaty H , Low LF , et al. Use of psychotropics in Sydney nursing homes: associations with depression, psychosis, and behavioral disturbances. Int Psychogeriatr. 2001;13(1):107‐120. 10.1017/s1041610201007505 11352328

[46] Lovheim H , Sandman PO , Kallin K , Karlsson S , Gustafson Y . Relationship between antipsychotic drug use and behavioral and psychological symptoms of dementia in old people with cognitive impairment living in geriatric care. Int Psychogeriatr. 2006;18(4):713‐726. 16879762 10.1017/S1041610206003930

[47] Macdonald AJ , Carpenter GI , Box O , Roberts A , Sahu S . Dementia and use of psychotropic medication in non‐'elderly mentally infirm' nursing homes in South East England. Age Ageing. 2002;31(1):58‐64. 11850309 10.1093/ageing/31.1.58

[48] Gerridzen IJ , Hertogh C , Depla MF , Veenhuizen RB , Verschuur EML , Joling KJ . Neuropsychiatric symptoms in people with Korsakoff syndrome and other alcohol‐related cognitive disorders living in specialized long‐term care facilities: prevalence, severity, and associated caregiver distress. J Am Med Dir Assoc. 2018;19(3):240‐247. 29079031 10.1016/j.jamda.2017.09.013

[49] Zarowitz BJ , O'Shea T , Nance M . Clinical, demographic, and pharmacologic features of nursing home residents with Huntington's disease. J Am Med Dir Assoc. 2014;15(6):423‐428. 24613270 10.1016/j.jamda.2014.01.010

[50] van Duinen‐van den IJCL , Mulders A , Smalbrugge M , et al. Nursing staff distress associated with neuropsychiatric symptoms in young‐onset dementia and late‐onset dementia. J Am Med Dir Assoc. 2018;19(7):627‐632. 29146222 10.1016/j.jamda.2017.10.004

[51] Kim H , Whall AL . Factors associated with psychotropic drug usage among nursing home residents with dementia. Nurs Res. 2006;55(4):252‐258. 16849977 10.1097/00006199-200607000-00005

[52] Wetzels RB , Zuidema SU , de Jonghe JF , Verhey FR , Koopmans RT . Course of neuropsychiatric symptoms in residents with dementia in nursing homes over 2‐year period. Am J Geriatr Psychiatry. 2010;18(12):1054‐1065. 21155143 10.1097/jgp.0b013e3181f60fa1

[53] Bauhuis R , Mulders AJMJ , Koopmans RTCM . The course of neuropsychiatric symptoms in institutionalized patients with young onset dementia. Aging & Mental Health. 2018;1‐6. 10.1080/13607863.2018.1531379 30499335

[54] Cipriani G , Lucetti C , Danti S , Nuti A . Apathy and dementia. Nosology, assessment and management. J Nerv Ment Dis. 2014;202(10):718‐724. 25265266 10.1097/NMD.0000000000000190

[55] Treusch Y , Majic T , Page J , Gutzmann H , Heinz A , Rapp MA . Apathy in nursing home residents with dementia: results from a cluster‐randomized controlled trial. Eur Psychiatry. 2015;30(2):251‐257. 24630745 10.1016/j.eurpsy.2014.02.004

[56] van der Spek K , Koopmans RT , Smalbrugge M , et al. Factors associated with appropriate psychotropic drug prescription in nursing home patients with severe dementia. Int Psychogeriatr. 2018;30(4):547‐556. 28931452 10.1017/S1041610217001958

[57] van der Spek K , Koopmans R , Smalbrugge M , et al. The effect of biannual medication reviews on the appropriateness of psychotropic drug use for neuropsychiatric symptoms in patients with dementia: a randomised controlled trial. Age Ageing. 2018;47(3):430‐437. 29432518 10.1093/ageing/afy001

